# Therapeutic success and failure in using miltefosine to treat dogs naturally infected with *Leishmania infantum*

**DOI:** 10.1590/S1984-29612024012

**Published:** 2024-02-12

**Authors:** Gustavo Gonçalves, Monique Paiva de Campos, Alessandra Silva Gonçalves, Fabiano Borges Figueiredo

**Affiliations:** 1 Laboratório de Biologia Celular, Fiocruz Paraná, Instituto Carlos Chagas, Curitiba, PR, Brasil; 2 Laboratório de Referência em Leishmaniase, Fiocruz Paraná, Instituto Carlos Chagas, Curitiba, PR, Brasil; 3 Pesquisadora independente, Campo Grande, MS, Brasil

**Keywords:** Canine visceral leishmaniasis, leishmaniasis treatment, parasite load, Leishmaniose visceral canina, tratamento de leishmaniose, carga parasitária

## Abstract

In urban environments, domestic dogs (*Canis familiaris*) are a major reservoir for the parasite *Leishmania infantum*. Miltefosine has been used as the standard treatment for canine visceral leishmaniasis in Brazil. However, therapeutic failures have been reported. In the present study, two dogs (CG03 and CG06) with a diagnosis of infection by *L. infantum* underwent two cycles of treatment with miltefosine (Milteforan™ - Virbac®). Analyses showed increases in the parasite load of both CG03 and CG06, even after treatment. The clinical score of CG03 dropped from 1 to 0 (after one round of treatment), such that this dog became asymptomatic. CG06 showed clinical worsening, such that its score increased from 1 to 2. After the second therapeutic round, the parasite load in CG03 was found to have decreased, but it was still higher than before drug treatment even though this dog was physically asymptomatic. There was no decrease in the parasite load in CG06 and there was clinical worsening. The clinical response of these dogs to the treatment differed, but the parasite load remained high in both cases, which poses a risk to public health, making it essential take measures to prevent the sandfly vector from accessing the dog.

## Resumo

Em ambientes urbanos, o cão doméstico (*Canis familiaris*) é um importante reservatório do parasito *Leishmania infantum*. Desde 2016, a miltefosina é utilizada como tratamento padrão para leishmaniose visceral canina no Brasil, porém falhas terapêuticas têm sido relatadas. Dois animais (CG03 e CG06) com diagnóstico confirmado de infecção por *L. infantum* foram submetidos ao tratamento com miltefosina (Milteforan™ - Virbac ®) durante dois ciclos. As análises mostraram aumento significativo na carga parasitária da pele de CG03 e CG06, mesmo após o tratamento com miltefosina. O “score” clínico do CG03 caiu de 1 para 0 (após uma rodada de tratamento), apresentando-se assintomático. Por outro lado, CG06 apresentou piora clínica, com sua pontuação aumentando de 1 para 2. Após duas rodadas terapêuticas, a carga parasitária diminuiu no CG03, mas ainda era maior do que antes do tratamento. O animal ainda se apresentava fisicamente assintomático. Não houve diminuição da carga parasitária de CG06, porém houve piora clínica. Sendo assim, o animal foi encaminhado para eutanásia. A resposta clínica dos animais foi diferente em resposta ao tratamento, mas a carga parasitária permaneceu elevada em ambos os casos, o que oferece risco à saúde pública, tornando essencial medidas para evitar o acesso do vetor flebotomíneo ao cão.

Leishmaniasis is a parasitic disease caused by protozoa of the genus *Leishmania* and transmitted by the bite of sandflies infected with the parasite. The most severe form of the disease in humans is human visceral leishmaniasis (HVL) (WHO, 2022)​, caused in Latin America by *Leishmania infantum* (syn = *Leishmania chagasi*) ​(Werneck, 2014)​. In urban environments, domestic dogs (*Canis familiaris*) are a major reservoir for the parasite (Herenio et al., 2014)​. Hence, controlling dog-to-dog and dog-to-human transmission forms the main challenge with regard to the epidemiology of the disease worldwide ​(Werneck, 2014)​.

Over the last 10 years, an average of 3000 new cases of HVL per year have been recorded in Brazil, and these have led to an average of 250 deaths per year. Over recent decades, the range of this disease has been expanding throughout the country, such that it has now reached hitherto unaffected areas such as the country’s southern region, and there is now an ongoing disease cycle in large cities. Despite a recent drop in the number of deaths, it is still a serious disease ​(Brasil, 2023)​.

Since 2016, miltefosine has been used as the standard treatment for canine visceral leishmaniasis (CVL) (Brasil, 2016). Until then, disease control in Brazil was based on euthanasia of seropositive animals, whether or not they were sick. Having the canine treatment available has been of great importance, since in most cases the therapy provides a clinical improvement and a reduction in the infectivity of the animal to sandflies ​(Nogueira et al., 2019)​. However, therapeutic failures have been reported both in monotherapy using this drug and in combined therapy with other drugs. Although the symptoms are reduced, the parasites are not completely eliminated from the dog ​(Andrade et al., 2011)​. Furthermore, cases of induced resistance have been observed ​(Gonçalves et al., 2021)​ and there have been reports of animals that remained susceptible to infection by the invertebrate vector despite reaching clinical cure​ (Chappuis et al., 2007)​.

For the present study, 20 dogs of different ages and breeds in the municipalities of Barra Mansa, Rio de Janeiro (RJ) (10 animals), and Campo Grande, Mato Grosso do Sul (MS) (10 animals), with clinical signs suggestive of CVL, were initially selected. The animals come from private owners and were selected through partner veterinarians who treat suspected cases of VL in different regions in both cities. After physical examination, a chromatographic immunoassay (DPP®, Bio-Manguinhos/Fiocruz) was performed on the animals to confirm the presence of infection. Additional samples were taken from the animals that tested positive in order to further confirm the infection by *Leishmania infantum* through qPCR (quantitative real-time PCR) and parasitological culture.

These dogs were assessed for the presence of the five most common presumptive signs of disease severity (alopecia, onychogryphosis, keratoconjunctivitis, body condition and lymphadenopathy). They were then classified in accordance with a semiquantitative scale (score) from 0 (absence of clinical signs) to 3 (severe signs), as presented by Campos et al. ​(2018)​.

For the qPCR process, a fragment of intact skin was collected using a punch of 3 mm in diameter. This sample was placed in a sterile bottle that was free from RNase and DNase, and it was stored at -20 °C until the time of the analysis. Species-specific primers were used in qPCR to confirm and characterize *L. infantum* infections. After DNA extraction using the DNeasy blood and tissue kit (QIAGEN®), the sample was amplified using the TaqMan® system on the StepOne™ platform (Applied Biosystems®). The TaqMan® MGB probe (FAM- 5'AAAAAAATGGGGTGCAGAAAT- 3'-NFQM-3GB) and the primers LEISH-1 (5'-AACTTTCTGGGCTCCGGGTAG-3') and LEISH-2 (5'-ACCCCCAGTT TCCCGCC-3') were designed to reach conserved regions of *L. infantum* kDNA ​(Francino et al., 2006)​. Three independent repetitions of the reaction were performed on each sample as described by Campos et al. (2017). An analysis on statistical differences between the different sample times was performed using the one-way ANOVA test.

For the parasitological culture, another skin fragment was collected and, additionally, bone marrow and lymph node aspirates were performed. These samples were stored under refrigeration (below 4 °C) in sterile saline containing antibiotics (Penicillin and Streptomicin, Sigma-Aldrich®) and antifungals (5-Fluorocytosine, Sigma-Aldrich®). After storage for 24 hours, the samples were seeded into a biphasic medium containing Novy-MacNeal Nicole (NNN) and Schneider (supplemented with 10% FBS). These cultures were examined under an optical microscope weekly for one month, to search for promastigote forms of the parasite.

Once infection by *L. infantum* had been confirmed using all the proposed methodologies (DPP®, qPCR and parasitological culture), treatment with miltefosine (Milteforan™ - Virbac®) was started, in accordance with the instructions in the drug packaging insert. The treatment consisted of daily doses of 2 mg/kg for 28 consecutive days, followed by a 4-month break (i.e. a typical round of treatment). The treatment rounds were repeated according to the need of each dog (parasite load levels), which were evaluated through new collections of biological material immediately before a new therapeutic cycle began. Concomitantly with the treatment with miltefosine, allopurinol (generic) was administered orally at a dose of 10 mg/kg/day, including during the 4-month interval between rounds of treatment with miltefosine.

The animals were kept throughout the treatment at their owners' residence and collared with a collar impregnated with deltamethrin (Scalibor®). The administration of medication to the dogs was carried out by the owners, in accordance with previous instructions of our team.

Among the 10 dogs that had been selected for the study in Barra Mansa (RJ), all of them showed positive results in the DPP® and qPCR tests. However, it was only possible to isolate the parasite in the culture from a single animal. This dog was subsequently withdrawn from the study by its keeper after it had undergone treatment with the drug for 28 days.

Among the dogs in Campo Grande (MS), the DPP® test was positive in the cases of eight of the ten animals selected for the study. The qPCR confirmed the presence of infection by *L. infantum* in the eight previously positive animals. However, isolation of the parasite in culture was only possible in the cases of two dogs: a two-year-old female of mixed breed (CG06) and a three-year-old male of Chow-Chow breed (CG03). The parasitological culture test enabled access to two isolates: MCAN/BR/19/CG06T0 and MCAN/BR/19/CG03T0. Following the three positive examinations, treatment with the drug was started.

It was not possible to stage all the animals due to a lack of initial examinations, but the dog CG06 did not present renal or hepatic alterations in biochemical tests. It had mild normocytic anemia and leukocytosis due to neutrophilia and lymphocytosis. Because of this, the clinical criterion for effectiveness of treatment was used, given that the aim of the treatment is to reach clinical cure.

**Figure 1:**
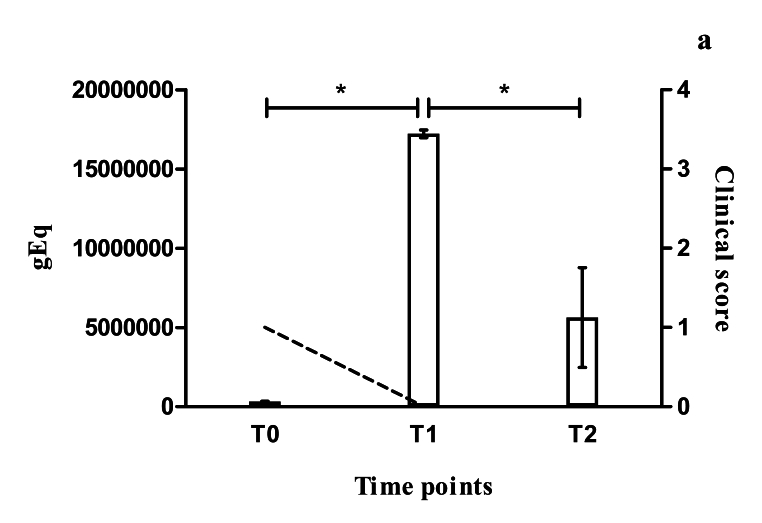
The bars show the parasite load observed in skin biopsies collected from dog CG03 before the start of miltefosine treatment (0) and after one (1) and two (2) rounds of miltefosine treatment (daily doses for 28 days). *P < 0.0001. The dashed line shows the evolution of the clinical score observed between the collection times.

**Figure 2:**
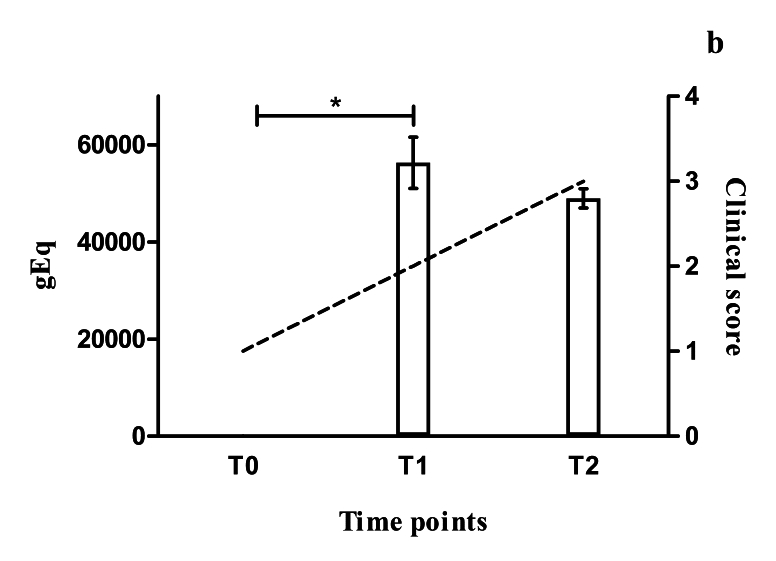
The bars show the parasite load observed in skin biopsies collected from dog CG06 before the start of miltefosine treatment (0) and after one (1) and two (2) rounds of miltefosine treatment (daily doses for 28 days). *P < 0.0001. The dashed line shows the evolution of the clinical score observed between the collection times.

After the 28-day treatment cycle and a four-month interval, new collections of biological material from both of these dogs (CG03 and CG06) were performed. Analyses on the parasite load using qPCR showed that significant increases in the parasite load had occurred in the skin of CG03 (F(2.6) = 805.9; P < 0.0001) and CG06 (F(2.6) = 264.1; P < 0.0001), even after treatment with miltefosine (Figures 1, 2). In contrast, the clinical score of CG03 dropped from 1 (before treatment) to 0 (after one round of treatment), such that this dog became asymptomatic (Figure 1). Meanwhile, CG06 showed clinical worsening, such that its score increased from 1 to 2 (Figure 2). The parasitological culture was only positive in CG06, resulting in the isolate MCAN/BR/19/CG06T1. Soon after sample processing and analysis on the results, both of these dogs underwent a new round of treatment because of the high parasite load that had been found.

After a 4-month interval, new collections were performed. After the second therapeutic round, the parasite load in CG03 was found to have decreased, but it was still higher than before drug treatment. The dog’s clinical score remained at 0, i.e. asymptomatic (Figure 1). On the other hand, there was no decrease in the parasite load of CG06 after the second round of treatment with miltefosine (P = 0.08), and there was clinical worsening, such that this dog’s clinical score increased from 2 to 3, the highest score on the scale used (Figure 2). The dog CG06 was found to present mild anisocytosis, persistence of normochromic normocytic anemia, monocytosis, hyperproteinemia, hypoalbuminemia and hyperglobulinemia. Furthermore, the parasite culture was again only positive in the case of CG06, resulting in the isolate MCAN/BR/19/CG06T2 (isolated after the second therapeutic round with miltefosine).

**Figure 3:**
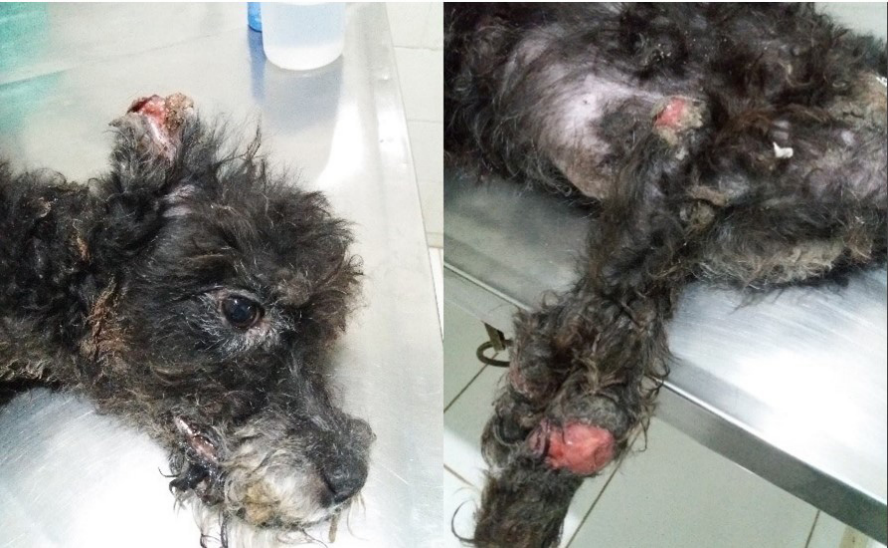
Dog CG06 after failure of treatment with miltefosine.

In the last clinical analysis, CG06 presented severe weight loss, alopecia, onychogryphosis, keratoconjunctivitis, lymphadenopathy, ulcerative skin lesions, hepatosplenomegaly, and apathy (Figure 3). Because of the progressive clinical worsening, even after two therapeutic rounds with miltefosine and daily doses of allopurinol throughout the period analyzed (approximately 10 months), along with the continuing high levels of parasite load (Figure 2), the case of CG06 was classified as a therapeutic failure. Consequently, and following the owner’s decision, this animal was sent for euthanasia.

The progressive increase in occurrences of therapeutic failure with miltefosine has led to great interest within the scientific community over recent years, regarding evaluation of the impact of *in vivo* and *in vitro* treatment with this drug on the acquisition and resistance mechanisms among parasites of the genus *Leishmania*. This has been demonstrated through studies on a Syrian hamster model and on mice and humans infected by *L. donovani* ​(Hendrickx et al., 2015; Srivastava et al., 2017; Van Bockstal et al., 2020)​.

Non-continuity of treatment with miltefosine is a practice often observed by veterinarians in the field. The main criterion usually adopted by dog owners with regard to deciding when to start and therapy using this drug is the dog’s clinical condition. However, this does not always faithfully reflect the animal’s parasitism level. This situation was observed in the present study, in which the dog CG03 had a much higher parasitic load on its skin than did CG06, which showed severe clinical signs, despite remaining clinically asymptomatic after drug treatment. This behavior of abandoning treatment is also one of the biggest obstacles preventing field researchers from carrying out studies of this nature.

The parasites isolated from CG03 showed slower growth and metabolism than did those isolated from CG06. This difference was probably due to genetic variability among the parasites in endemic areas. Existence of such differences possibly made isolation of the parasite through culture difficult in the first collection and impossible in subsequent collections.

Although greater severity of the clinical picture of the disease have been correlated with high parasite loads ​(Chagas et al., 2021)​, it is already known that physically asymptomatic dogs can transmit the parasite to the invertebrate vector ​(Laurenti et al., 2013). This possibly helps maintain disease transmission in endemic areas ​(Moshfe et al., 2009)​, since the main factor in determining a dog's capacity for disease transmission is the level of parasitism in its skin ​(Borja et al., 2016)​.

Therapeutic failures of miltefosine, as observed in CG06, have previously been reported from therapy with this drug ​(Proverbio et al., 2014). These may be related to several factors, ranging from natural resistance among strains circulating in Brazil ​(Espada et al., 2021)​ to the animal's immune response ​(Giunchetti et al., 2019)​. The latter factor is known to be fundamental for determining whether the prognosis for the disease in dogs is good (CG03) or poor (CG06).

In the context of intensive use of a drug that is not 100% effective, such as miltefosine ​(Manna et al., 2015)​, where successive rounds of treatment do not ensure complete elimination of the parasite from the animal's body ​(Manna et al., 2009),​ these findings may have serious implications for maintaining disease transmissibility in endemic areas and may lead to risks to animal and human health. For these reasons, treatment of the disease is always done together with the use of repellents on both infected and non-infected dogs and use of insecticides in homes in endemic areas. All the dogs involved in the present study used a collar impregnated with deltamethrin (4%) (Scalibor®) throughout the study period.

There is therefore an urgent need for further studies to determine the real effectiveness of treatment with this drug in the field in Brazil, and to determine the influence of natural resistance among circulating strains and of genetic factors relating to the dog that may be involved in therapeutic success. Measures to control the spread of vectors are equally urgent, given that treatment and euthanasia have not been shown to be satisfactory measures (Romero & Boelaert, 2010)​. Another important point to be analyzed is the impact of treatment with miltefosine on generation of resistant parasites, as previously observed by our research group ​(Gonçalves et al., 2021)​.

The presented case report describes partial clinical results from dogs naturally infected with *L. infantum* and treated with miltefosine. The limitations of this study were the small number of animals analyzed, resulting from the difficulties inherent in this type of follow-up study, and the fact that the disease was only partial staged, based mainly on physical evaluation and some biochemical and hematological examinations.

In conclusion, the clinical response to treatment of these animals differed, but the parasite load remained high in both cases. This finding demonstrates that the maintenance of infected animals can pose risks to public health, making it essential the need for measures to protect animals against disease vectors and for veterinary monitoring throughout the treatment with the drug.
